# Solitary Spinal Epidural Metastasis from Prostatic Small Cell Carcinoma

**DOI:** 10.1155/2016/4728343

**Published:** 2016-06-16

**Authors:** Kyung Ryeol Lee, Young Hee Maeng

**Affiliations:** ^1^Department of Radiology, Jeju National University Hospital, Aran 13gil 15 (Ara-1-Dong), Jeju-si, Jeju Special Self-Governing Province 63241, Republic of Korea; ^2^Department of Pathology, Jeju National University Hospital, Aran 13gil 15 (Ara-1-Dong), Jeju-si, Jeju Special Self-Governing Province 63241, Republic of Korea

## Abstract

Solitary, spinal epidural metastasis (SEM) that is not related to vertebral metastasis is very rare. And solitary SEM from prostatic cancer is rarely found in previously published reports. However, it is clinically significant due to the possibility of neurologic dysfunction, and it can be assessed by MRI. In this report, we show a case of solitary SEM arising from prostatic small cell carcinoma detected by MRI.

## 1. Introduction

Metastasis to the spinal nervous system can be classified as intramedullary, epidural, and leptomeningeal metastases. Most of the epidural metastases in the spine are associated with vertebral metastases [1]. Solitary spinal epidural metastasis (SEM) which is not related to vertebral metastasis is very rare. However, the diagnosis of solitary SEM is very important because it can result in neurological complications. MRI is the imaging modality of choice for the assessment of solitary SEM [2]. We report a rare case of solitary SEM from prostatic small cell carcinoma detected by MRI.

## 2. Case Presentation

A 68-year-old man diagnosed two years ago with adenocarcinoma of the prostate came to our hospital because of low back pain. At that time, he had already received hormone therapy for two years. His low back pain started three years ago, although it became severely aggravated two weeks ago before visiting the hospital. His past medical history included hypertension, although he had no history of another cancer except for prostate cancer. On physical examination, hypoesthesia was detected in the L5 dermatome. His laboratory findings were within a normal range including his PSA level.

**Figure 1:**
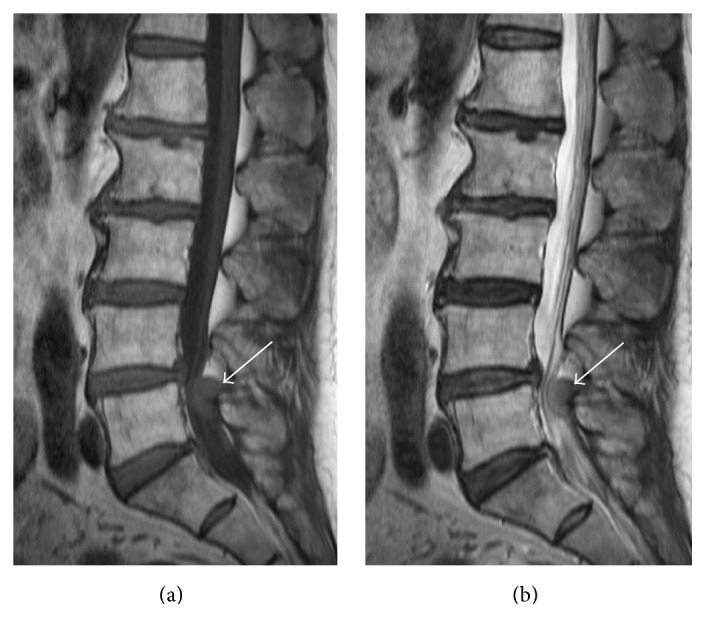
Sagittal T1-weighted MR image (a) and sagittal T2-weighted MR image reveal solitary spinal epidural mass (arrow) showing slightly high signal intensity as compared with signal intensity of skeletal muscle.

**Figure 2:**
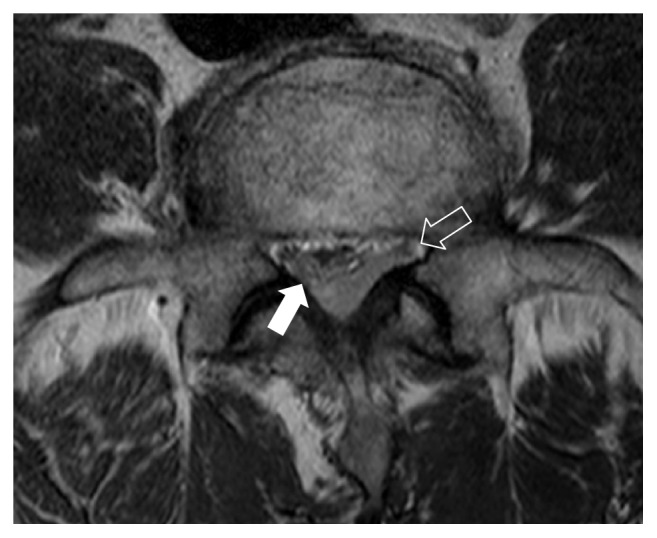
Axial T2-weighted image in upper body level of L5 reveals compression of thecal sac (solid arrow) and left S1 nerve root (open arrow).

**Figure 3:**
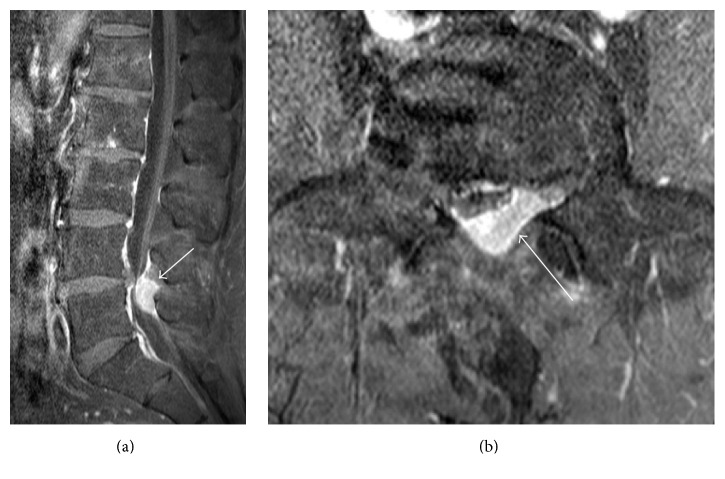
Sagittal (a) and axial (b) gadolinium-enhanced T1-weighted MR images with fat saturation show intense enhancement of solitary spinal epidural mass (arrow).

**Figure 4:**
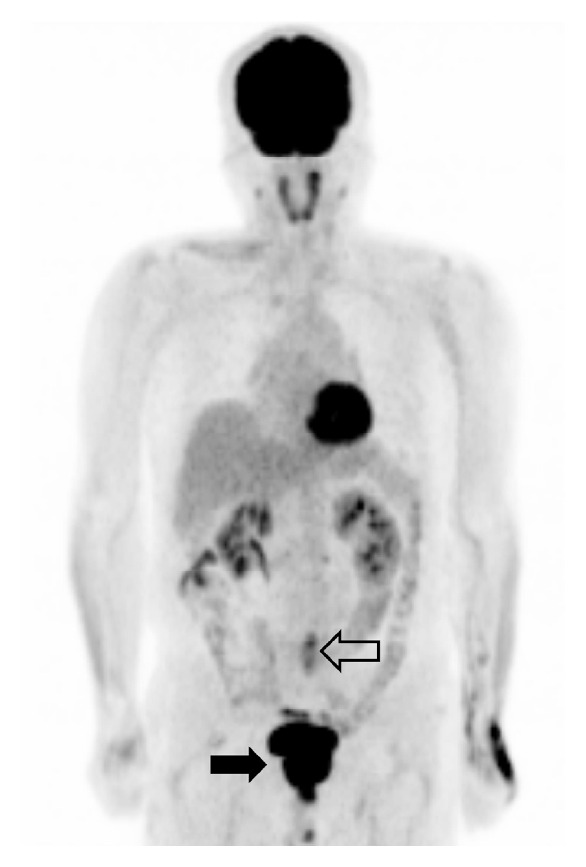
Coronal FDG PET image performed after surgery shows no abnormal uptake suggesting malignancy or metastatic tumor in the body except intense uptake due to prostate cancer and urine in the pelvis (solid arrow) and shows focal increased uptake due to postoperative change in L4/5 level (open arrow).

**Figure 5:**
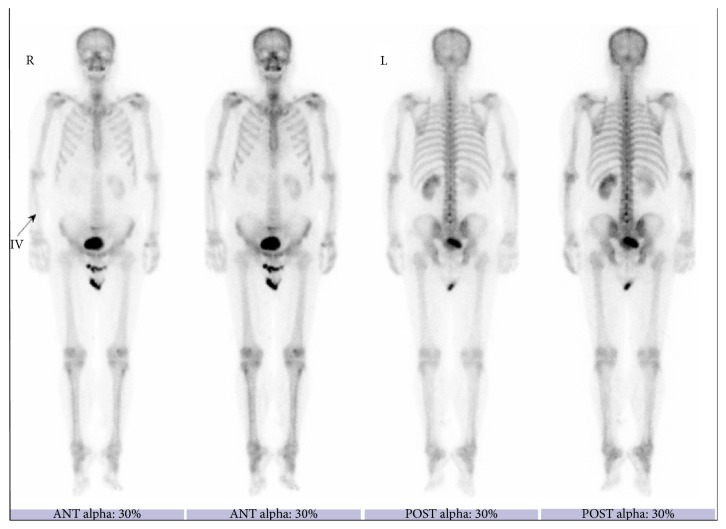
Bone scintigraphy performed after surgery shows no intense uptake suggesting bone metastasis.

As his attending urologist suspected that his chronic low back pain was due to a degenerative disease such as spinal stenosis, he recommended MR imaging which showed a space-occupying lesion in the posterior epidural space at the L4/5 level. On T1-weighted and T2-weighted images, the lesion showed slightly high signal intensity compared with that of the skeletal muscle (Figure 1). The mass had an irregular shape and resulted in a thecal sac and left L5 nerve root compression (Figure 2). The lesion did not develop as an outgrowth and with invasion of vertebral body metastasis to the epidural space. In addition, gadolinium-enhanced, fat-suppressed, T1-weighted images revealed intense and heterogeneous enhancement (Figure 3). Because the patient had prostate cancer, we suspected the possibility of epidural metastasis. However, the differential diagnosis included other space-occupying lesions such as a sequestrated lumbar disc, complicated synovial cyst, hematoma, and malignant lymphoma. In other imaging studies, including a PET/CT and bone scintigraphy, we could not find any other systemic metastasis (Figures 4 and 5), and, therefore, a consulted neurosurgeon planned and performed excision of the epidural mass lesion.

**Figure 6:**
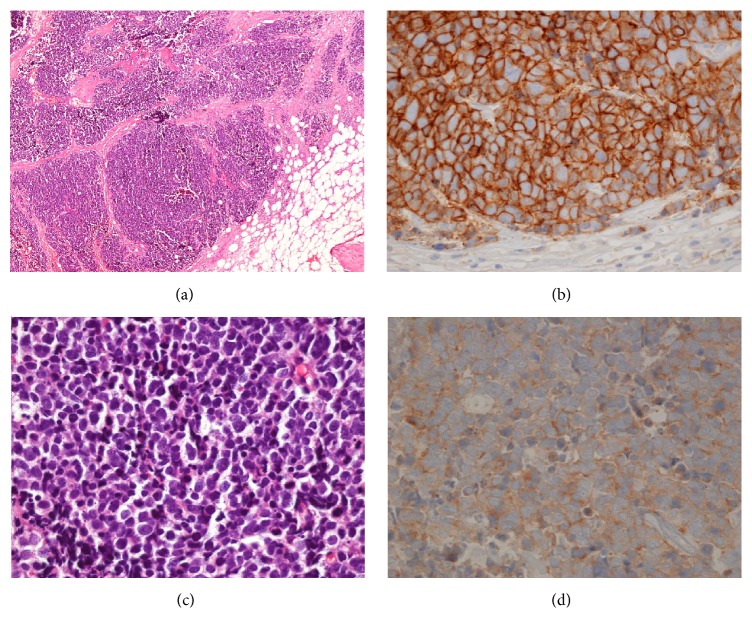
Photomicrographs showing diffuse infiltration of small anaplastic cells ((a) H&E, ×40; (b) H&E, ×400) and immunohistochemical reactivity of tumor cells for CD56 ((c) ×400) and synaptophysin ((d) ×400).

Eventually, the mass was removed by en bloc resection. According to the intraoperative findings, the tumor was dissected well in the dura. On the gross pathology examination, the excised tumor, measuring 2.0 × 1.5 × 0.8 cm, had a grayish white color. On the microscopic pathology examination, the tumor was proven to be a small cell carcinoma and immunohistochemically the tumor was positive for CD56, synaptophysin, and CK, but with negative PSA (Figure 6). The pathologist indicated that the immunohistochemical findings suggested the possibility of neuroendocrine carcinoma of the small cell type and that the primary site of the tumor cells (adenocarcinoma) could not be identified. Therefore, we cannot be sure that the tumor was a solitary spinal epidural metastasis (SEM) of the prostate cancer.

However, four months following excision of the solitary SEM, the patient underwent transurethral resection of the prostate (TURP) at an outside hospital due to his obstruction symptoms and nocturia, and the resected prostate cancer was histologically determined to be small cell carcinoma. Therefore, we concluded that the patient's prostate cancer was mixed type and that the spinal epidural mass was a solitary SEM.

The patient underwent L4-L5 postoperative radiotherapy with 30 Gy in 10 fractions for two weeks, after which palliative chemotherapy and radiation therapy for residual prostate cancer followed. Following excision of the solitary SEM of the L4/5 level, the patient's symptoms were slightly relieved. However, progression of the residual prostate cancer and lung metastasis were subsequently detected and the patient died 11 months after excision of the solitary SEM.

## 3. Discussion

Metastasis to the spinal nervous system includes intramedullary metastases, epidural metastases, and leptomeningeal metastases [3]. These spinal nervous system metastases are medical emergencies resulting in neurological dysfunction for which a rapid diagnosis and therapeutic intervention are required [1]. Usually, spinal epidural metastasis (SEM) arises from spinal vertebral body metastases, and the vertebral body is primarily involved because of its highly vascular red marrow. As a tumor grows, bone resorption due to osteoclast activating factors and prostaglandins, as well as bone destruction, occurs. Subsequently, SEM develops as an outgrowth and invasion of vertebral body metastasis to the epidural space [1, 3].

Lung, breast, and prostate cancer each account for 15–20% of all cases of SEM, and non-Hodgkin's lymphoma, renal cell cancer, and multiple myeloma account for 5–10% of these cases, and the remainder of the cases of SEM are colorectal cancers, sarcomas, and unknown primary tumors. This tendency is closely related to the tendency of the type of tumor to metastasize to bone and the spine [1].

Solitary SEM that is not related to vertebral metastasis is very rare. We have no concept regarding the incidence or the type of the primary tumor of solitary SEM based on our literature review. There have been three reports regarding solitary SEM cases. Madden et al. reported thoracic SEM of Merkel cell carcinoma in an immunocompromised patient. In this patient, SEM represented a bilobed, epidural mass extending from T6 to T8 with extension into the left T6/T7 neural foramen, and caudal extension into the left T8/T9 neural foramen as seen on precontrast CT images. As this patient had remaining metal fragments in his right eye following previous surgery, MRI could not be performed. The patient presented with mid-thoracic back pain radiating around the trunk, and this symptom was relieved after his surgery [4]. Gupta et al. published a report regarding SEM from lung carcinoma. In this patient, SEM presented with focal, enhancing, epidural, and soft-tissue thickening in the right side of the T12-L1 level, as seen on MR imaging. The authors did not mention the signal intensities of the lesion seen on T1- and T2-weighted images. This patient complained of back pain, and her symptom was relieved after she underwent radiotherapy [5]. Brown et al. reported SEM in an endometrial carcinoma patient. In this patient, SEM appeared as a peripheral enhancing epidural mass located at the L5 level on MR images. The authors also did not mention the signal intensities of the lesion, as seen on T1- and T2-weighted images. The patient complained of back pain and paresthesia radiating in her lower legs, and she underwent surgical resection and postoperative radiotherapy [6]. Based on these case reviews, our reported case is very important because it reveals both the signal intensities on T1- and T2-weighted images and the enhancement character of solitary SEM in a prostate cancer patient.

The imaging differential diagnosis of epidural, space-occupying lesions includes both tumor lesions and nontumorous lesions mimicking tumors. Tumor lesions include metastasis, schwannoma, and malignant lymphoma, while tumor-mimicking lesions include sequestrated intervertebral disc, hemorrhagic synovial cyst, hematoma, and abscess [7]. When we discover an epidural, space-occupying lesion on MRI, it is very difficult to distinguish whether it is a tumor or tumor-mimicking lesion. The signal intensities on T1- and T2-weighted images and the enhancement pattern help us to differentially diagnose epidural, space-occupying lesions. However, there is limited suspicion regarding the specific disease. Furthermore, reported cases of sequestrated disc and hemorrhagic synovial cyst showed a mass effect and intense enhancement and mimicked tumor occurring in the epidural space of the spine [7, 8]. Therefore, based on our literature review, it is important to detect a lesion in the epidural space using MRI before neurologic complications occur and to surgically excise the lesion in order to obtain an accurate diagnosis and initiate appropriate treatment. The signal intensities on T1- and T2-weighted images and the enhancement pattern of epidural, space-occupying lesions are described in Table 1 [7, 9, 10].

Our reported case was solitary SEM of prostate cancer of the small cell type. The majority of prostate cancers are acinar adenocarcinomas. Histological variants of prostate cancers can be defined as acinar adenocarcinoma and nonacinar carcinoma variants. The nonacinar carcinoma variants account for approximately 5–10% of the carcinomas that occur in the prostate. These include sarcomatoid carcinoma, ductal adenocarcinoma, urothelial carcinoma, basal cell carcinoma, and small cell carcinoma, in other words, neuroendocrine tumors. Small cell carcinoma developing in the prostate is a rare and very aggressive tumor, which frequently presents with disseminated disease. The incidence of prostate small cell carcinoma ranges from 0.3% to 1.0% in all prostate cancers. Prostatic small cell carcinoma shows different clinical features from those of prostatic acinar adenocarcinoma. Distinguishing clinical features include a lower percentage of men who present with an elevated serum PSA level at advanced stages of prostate cancer, poor hormonal responsiveness, and a short patient survival time. Most patients are 65–72 years old, and the most frequent presenting symptoms arise from bladder outlet obstruction and disseminated disease. One-third to two-thirds of patients with prostatic small cell carcinoma show an elevated serum PSA level, which could be due to an admixed adenocarcinomatous component [11–13].

Approximately half of the cases of prostatic small cell carcinomas are mixed tumors with conventional prostate cancer. The grade as well as extent of the acinar adenocarcinoma component in mixed small cell adenocarcinoma in the prostate is variable. In most patients, if adenocarcinoma is diagnosed earlier than small cell carcinoma, the histologic grade of the adenocarcinoma portion is low-grade. In metastatic lesions derived from primary, mixed, small-cell carcinoma-adenocarcinoma, the small cell carcinoma element is sometimes seen, although both components can be found [11].

The diagnosis of solitary SEM begins with clinical suspicion in a known cancer patient complaining of new onset pain. And the solitary SEM is assessed by imaging modalities including plain radiographs, myelography, CT/CT myelography, and MRI. MRI is the method of choice for the diagnosis of solitary SEM. It is most useful and efficient that when a physician performs MRI, MRI of the whole spine should be performed. CT myelography remains an alternative method when MRI is unavailable or cannot be used because of uncontrolled pain, patient size, implanted metallic objects, the inability to lay flat during the exam time, or severe claustrophobia. The recommended treatment varies and includes surgical treatment, radiation therapy and chemotherapy, corticosteroids, or bisphosphonate [1, 3].

## Figures and Tables

**Table 1:** The differential diagnosis according to MRI findings of the epidural space occupying lesion.

	T1WI	T2WI	Gd enhancement
Metastatic tumor	Low	High	−~+
Schwannoma	Low~iso	High	++
Lymphoma	Low~iso	High or low	+
Sequestrated intervertebral disc	Low	High	+ (rim enhancement)
Hemorrhagic synovial cyst	High	High or low	−/+
Hematoma	Variable	Variable	−
Abscess	Low~iso	High	+ (rim enhancement)
